# A Scoping Review and Narrative Synthesis Comparing the Constructs of Social Determinants of Health and Social Determinants of Mental Health: Matryoshka or Two Independent Constructs?

**DOI:** 10.3389/fpsyt.2022.848556

**Published:** 2022-04-14

**Authors:** Fritz Handerer, Peter Kinderman, Matina Shafti, Sara Tai

**Affiliations:** ^1^School of Health Sciences, University of Manchester, Manchester, United Kingdom; ^2^Department of Psychological Sciences, University of Liverpool, Liverpool, United Kingdom

**Keywords:** mind-body, structural factors, comparing theories, physical versus mental health, social determinant, public mental health

## Abstract

**Background:**

Many health research policies invoke the construct of Social Determinants of Health, and more recently the construct of Social Determinants of Mental Health. While frequently referred to in the literature, it is unclear how these constructs relate to each other. Some commentators conceptualise the Determinants of Mental Health as a subgroup of the Determinants of general Health and others describe the Determinants of Mental Health as an autonomous construct. The current review investigates the relationship between both constructs.

**Methods:**

Comprehensive literature searches were conducted for both constructs separately within seven electronic databases. A template analysis was conducted to compare the conceptualisations of the Social Determinants of Health and the Social Determinants of Mental Health.

**Results:**

Of 4250 search results, 50 papers (25 for each construct) fulfilled our inclusion criteria and were incorporated into a narrative synthesis. Discussions of the Social Determinants of both general and Mental Health listed the same determinants. Both constructs were conceptualised on multiple levels and factors. Stress and health behaviour were also described as mediators for both constructs. The constructs differed, however, with respect to two components of their aetiologies and epistemologies. First, the causal mechanisms invoked for the Determinants of general Health followed predominantly direct pathways, in contrast to indirect pathways for the Social Determinants of Mental Health. Second, the Social Determinants of Mental Health were reported to influence mental health mediated through individuals’ perceptions and appraisal processes. Appraisal processes were considered of far less relevance in the construct of Social Determinants of Health.

**Conclusion:**

The constructs of Social Determinants of Health and Social Determinants of Mental Health align in many respects but differ on important aetiological and epistemological grounds. Similar social factors are considered important, but whereas physical health conditions are primarily conceptualised to be driven by objective realities, mental health is explained mainly in terms of perception of these realities. This differentiation between physical and mental health is in line with a modern understanding of mind-body-dualism, the naturalistic dualism after Chalmers. Differentiating the Social Determinants of Mental Health from the Social Determinants of Health might bear relevance for policy making and research.

## Introduction

The Social Determinants of Health (SDH) are defined as “the conditions in which people are born, grow, live, work and age” (1). Health is in this context not clearly defined but means general health with a pronounced focus on physical health (2). The Social Determinants are seen as the driving forces of health inequalities between and within societies (3). Subsequently, the concept of SDH represents a shift from a traditionally individualistic perspective of medicine toward an understanding of health as being significantly shaped by structural factors (4). The World Health Organisation (WHO) contributed to this perspective shift by establishing the *Commission on the Social Determinants of Health* which delivered its final report in 2008, stressing the significance of social conditions for health. Further evidence of the broad acceptance of the SDH construct can be found in highly impactful publications (5, 6) and several political campaigns (7, 8). In the last years, demand for improved SDH data at a patient level (e.g., 9) has led to the development of several assessment tools for clinical practice (1, 10). Recently, an overview of such tools revealed 15 commonly assessed domains, such as safety, housing, and food insecurity (11). Application of these assessment methods have the potential to deepen knowledge about the causes and prevention of many illnesses (12).

Starting around 2010, the construct of Social Determinants of Mental Health (from now on shortened to SDMentH, to differentiate them clearly at an acronym level from the Social Determinants of Health; SDH) began to gain the attention of researchers. Examples of SDMentH are low income, social support, and education (13). The SDMentH are considered to be generally less well conceptualised than the SDH (2). Some researchers conceptualise the SDMentH as an autonomous construct (13, 14), while others understand mental health as one Social Determinant of Health and thereby the SDMentH as one aspect of the more general SDH, the SDMentH as a conceptual Matryoshka doll nested within an encompassing bigger figure of SDH (15, 16). There is currently no validated method of assessing the SDMentH, although many researchers demand that clinicians obtain and record information about patients’ social condition that may have contributed to mental health problems (17, 18).

To date, the constructs of SDH and SDMentH have not been compared. This is a significant shortfall on three accounts. First, an unclear delineation between SDH and SDMentH hinders improved aetiological understanding. Using the two terms interchangeably (19) implies that the same social determinants affect physical health in the same way as mental health. Understanding SDMentH as an autonomous construct on the other hand proposes differing determinants and pathway mechanisms (2). These contradictory positions regarding mental and physical health represent a vagueness that has been described as an impediment for aetiological progress (20). Second, if the relationship between SDH and SDMentH is unclear, it remains questionable whether the same interventions work for both constructs. Therefore, there is a health care imperative for sound theoretical conceptualisations because prevention programs are more effective when they are theoretically informed (21, 22). Third, lack of clarity over the relationship between both constructs means that assessment tools for SDH cannot simply be used to assess the social conditions most relevant for the development of mental health problems. This is because, using an SDH assessment tool to assess the SDMentH could lead to theoretically inconsistent data collection if the constructs significantly differ from each other. Theoretically inconsistent data collection and clinical record keeping can lead to critical misinterpretation and thereby inappropriate conceptualisation of constructs (23). To clarify the relationship between the constructs of SDH and SDMentH, therefore, this scoping review addressed the following questions:

1.) **What are considered to be determinants in the conceptualisations of Social Determinants of Health and Social Determinants of Mental Health?**
2.) **Which epistemological assumptions underpin both constructs (SDH and SDMentH)?**
3.) **What does the literature tell us about potential pathways from Social Determinants of Health and Social Determinants of Mental Health to specific experiences of individuals?**
4.) **What are the similarities and differences between SDH and SDMentH in relation to these questions?**

## Methods

### Search Strategy

This scoping review was conducted in accordance with the preferred reporting items for systematic reviews and meta-analyses, extension for scoping reviews (PRISMA) (24). In line with existing guidelines, we conducted a scoping review rather than a systematic review, because of the main research interest in clarifying two constructs (25). A protocol was pre-registered on the OpenScienceFramework (osf.io/796gh). We systematically searched seven electronic databases: Embase, Psycinfo, CINAHL, Pubmed, Sociology Abstracts, Proquest and Web of Science, for papers published in English or German before March 2020. Publications outside of scientific journals (grey literature) were searched for within Proquest, gov.uk and Nice evidence. This selection of grey literature sources was in line with a review that had a similar literature target (26). Eligible papers were hand-searched for references of other relevant papers.

### Inclusion and Exclusion Criteria and Search Terms

#### Social Determinants of Health

The literature on SDH is so extensive that the scope of this systematic literature search had to be constrained. We focused on papers that deal with SDH-assessment tools as measuring presupposes a good understanding of the object of interest, and we only included those papers that explicated this underlying understanding. Moreover, measuring itself is arguably a form of definition (27). Furthermore, theoretical underpinnings of assessment tools are particularly critical (28). An exhaustive analysis of the existing theoretical papers on the SDH would have been infeasible in face of the profuse literature. Thus, papers were included if they dealt with the generation of an assessment method, or with the theoretical underpinnings of developing SDH-assessment methods, of one or more determinants. Inclusion was restricted to papers which used the term “social determinants of health” and defined this construct. Peer reviewed articles were included, along with editorials, dissertations, and governmental and NGO publications. Posters and meeting abstracts were excluded, but the authors were contacted for any publication providing a broader contextualisation. Reviews of existing assessment tools were excluded to avoid repeated analyses of the same tools, but the reviews were used to reveal tools that were subsequently followed up for suitable publications. No restrictions were made regarding the methodology used in the papers, thus qualitative, quantitative, and mixed-method research was considered. A tabulated overview of the inclusion criteria can be found in the Supplementary Material 1. These criteria resulted in the following search terms: [(social determinant* of health OR non-medical health-related social need* OR structural determinant* of health OR wider determinant* of health) AND variants of assessing (screen* OR assess* OR check* OR measur* OR tool OR question* OR indic* OR scale* OR record*)]. Search terms and strategy were in line with recent reviews on the existing screening tools for the social determinants of health (10, 29–31).

#### Social Determinants of Mental Health

There is less literature on the construct of Social Determinants of Mental Health than the Social Determinants of Health. A preliminary systematic search for assessment methods specific to the SDMentH revealed no relevant results. We therefore included literature relating to theoretical groundwork, without limiting the search more. A well-grounded theory is the condition of construct validity (32) and thus the prerequisite of any assessment with good psychometric abilities. Moreover, the literature on SDMentH is far less extensive than the literature on SDH, which made this focus feasible for the SDMentH-literature. Papers were therefore included if they used the term “Social Determinants of Mental Health” and could serve as the basis for an assessment-tool-development to ensure construct validity. Included paper were required to clearly elaborate on an original framework, i.e., describe pathway mechanisms and determinants. Peer reviewed articles were included, along with dissertations, editorials, and governmental and NGO publications. Posters and meeting abstracts were excluded. As with the literature of SDH, no restrictions were made regarding methodology. All inclusion criteria are listed in the Supplementary Material 1. In generating the search terms, we followed Booth and Carroll’s guidance (33) on the systematic search for theory. This led to the following terms: [(Social determinant*” OR “SDMENTH” OR “SDMOH” OR “social need*”) AND (“mental health” OR “wellbeing” OR “well-being” OR “mental disorder*” OR “mental illness*” OR “mental” OR “psyc*” OR “life satisfaction” OR “quality of life”) AND (model*” OR “theor*” OR “concept*” OR “framework*”)]. [A list of the inclusion and exclusion criteria can be found in in Supplementary Material 1].

### Selection and Extraction

Two reviewers (FH and MS) independently reviewed the first 50 citations in order to test the reliability of the inclusion/exclusion criteria. Discrepancies between reviewers were resolved collaboratively following full text screening and with the advice of second and fourth reviewers (PK and ST). The agreement rate between both primary reviewers was 96% (SDH) and 98% (SDMentH) for the title/abstract screening; and 98% (SDH) and 84% (SDMentH) for the full text-screening. These good to excellent agreement rates were understood as confirmation of the reliability of the criteria (32). The primary investigator continued screening and extracting single handedly in line with the AMSTAR 2 instrument that appraises the quality of systematic reviews (34). Data on the four research questions, (the considered determinants, epistemological underpinnings of the constructs, proposed pathway mechanisms, and both constructs in comparison to each other) were collected in two tables, one table for each construct, next to basic information on the publication, such as name of authors and publication, year of publication, origin, research discipline and publication type.

**FIGURE 1:**
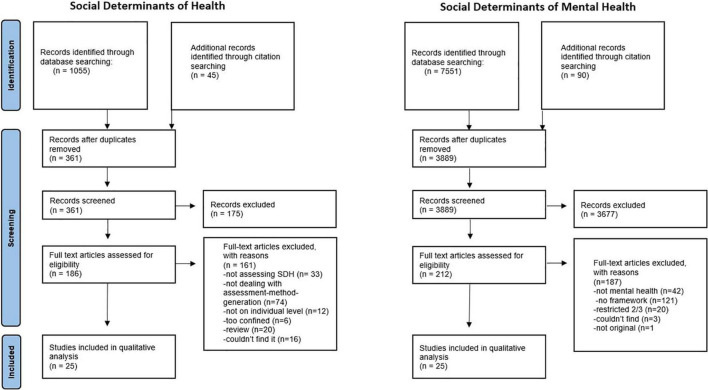
Prisma diagrams.

Figure 1 shows the PRISMA flow diagrams for both literature searches. The searches on the seven databases for the SDH literature yielded 1055 papers, 361 after removal of duplicates. One author (FH) scanned all the resulting papers. Papers that were excluded on the abstract/title screening level most commonly dealt with irrelevant subjects that shared the same acronym (e.g., Subdural hematoma, or Synchronous Digital Hierarchy) or with the assessment of psychosocial aspects of certain diseases like Parkinson or cancer. 25 papers fulfilled the inclusion criteria and were consequently included in the analysis. The searches for the SDMentH literature yielded 7751 results, 3889 after duplicates removal, which have all been screened by the primary investigator. Most papers that were excluded at the title/abstract screening stage reported specific relationships and referred only cursorily to social contextual factors. Other excluded papers dealt with determinants of outcomes that were not related to mental health (e.g., second language acquisition, or Type 2 diabetes in youth etc.) 25 of these papers met the inclusion criteria and have therefore been included in the analysis.

### Synthesis and Analysis

Synthesis and analysis differed for the four questions.

Question 1, the factors identified as social factors determining health and mental health, were compiled as they appeared in the literature. Subsequently they had to be summarised on a less concrete level to make them more easily comprehensible and comparable between the different papers and between the two constructs. To this purpose we used the domain classification of a recent review of SDH-assessment methods as a template (11). Moen and colleagues analysed nine commonly used SDH-assessment tools and identified 15 domains (11). In our analysis we initially allocated the extracted determinants to these 15 domains and added further domains where needed.

Questions 2, 3, and 4 required a comparison of theories within a scoping review. The issue of comparing theories in systematic and scoping reviews has gained prominence more recently, particularly in public health (35–37), though there is yet no consensus on appropriate methodology. Our analysis was guided by a method developed by Pound and Campbell (38). Theory analysis and synthesis rested on the following steps of Pound and Campbell’s model:

“1. Synthesis preparation: the clarification of existing theories, the extraction of what is useful, plausible and relevant to the purpose of the synthesis. 2. Synthesis: making theories comparable by breaking them down into simple propositions and rendering them abstract; comparison of the theories for points of convergence and divergence; bringing together those aspects that converge” (38).

We conducted a template analysis in order to reveal the “simple propositions” Pound and Campbell recommend. Template analysis is a particularly flexible form of thematic analysis, conducted in the following steps: data familiarisation; coding; organising these codes into themes; development of a coding template on the basis of a data sample; application of this template to a wider sample of the data; adaption of the template to better cover the data; application of the final template to the whole data set (39). Template analysis is considered to be a useful and systematic method (39, 40), used previously in the context of Pound and Campbell’s model (41).

### Results

#### Included Papers

##### Social Determinants of Health

The 25 included articles relating to Social Determinants of Health consisted of: five papers reporting on the development of a tool (42–46), two papers reporting the development and validation of a tool (47, 48), eight papers reporting the development of a tool and a feasibility study (49–56), one implementation and toolkit (57), three opinion articles (viewpoints/commentaries/perspectives) on the general task of developing an assessment tool (58–60), one guideline on the general task of assessment tool development (61) and two discussion papers on this task (62, 63). The majority of papers dealt with multiple determinants, with the exception of articles focusing on gender (63), religious social capital (48), food insecurity (47) and poverty (50, 62). Three papers were published by NGOs (the American Association of Pediatrics, the National Association of Community Health Centers and Health Begins), and one by a governmental institution (42), while the remaining 21 papers featured in peer reviewed journals. Twelve of the 25 included papers came from a family medicine or paediatric background (46, 50, 52–56, 59–61, 63, 64). The remainder was distributed to social work (43, 51), health research (44, 45, 47), psychiatry (45, 48), pharmacy (47), public health (58, 64), health economics (62) and social medicine (49, 58). All first authors were from the United States, except for one paper from Australia (45), one from South Africa (62), and three from Canada (50, 52, 63). [A list of all included papers in the Social Determinants of Health section can be found in Supplementary Material 2].

##### Social Determinants of Mental Health

The 25 identified articles reporting on the Social Determinants of Mental Health included seven papers providing an unspecified overview of theories with accompanying policy recommendations (65–71) or accompanying propositions of how the research field should develop (72, 73), or how practitioners should incorporate the theory in their work (74). Four papers included a proposition of a model (75–78) and different forms of reviews (critical review, systematic review of reviews, and an unspecified review) (76, 79, 80). Five papers were, by and large, quantitative studies (81–85). The majority of included papers were peer reviewed journal articles (17 out of 25). Four book chapters were included (67–69, 77), three doctoral dissertations (82, 84, 85) and one governmental report (71). The scientific backgrounds of the authors were rather diverse, with the majority in the field of psychiatry (65, 69, 70, 72, 74, 76, 83), and psychology (78, 80, 83, 84), but also public health (68, 73, 75, 86), global mental health (65, 68–70), sociology (85), neurology (67), social work (82, 87), nursing (88), and epidemiology (89). Eight of the 25 first authors were from the United States (70, 72, 74, 78, 82, 83, 85, 89), five from Australia (71, 73, 75, 81, 86) and four from the United Kingdom (67, 68, 87, 88). Furthermore, the majority discussed loosely defined Social Determinants of Mental Health for an unspecified population. However, two papers confined the population to refugees (77, 79), one to men (65), and one restricted the scope to severe mental illness (85). Others dealt only with certain determinants, namely democracy (86), racial and ethnic disparities (78), and globalisation (80). Two papers looked at the SDMentH from a specific discipline perspective: mental health nursing (88) and social work (87). [A list of all included papers in the Social Determinants of Mental Health section can be found in Supplementary Material 3].

## Results

The results of the analysis of the 50 included articles will be presented in order of the questions. Questions 1 to 3 will firstly be answered for the construct of Social Determinants of Health and subsequently for the Social Determinants of Mental Health. The answer to question 4 will compare both constructs in an overview.

**Question 1**: **What are considered to be determinants in the conceptualisations of Social Determinants of Health and Social Determinants of Mental Health?**

### Social Determinants of Health

We used the findings of a recent review on assessment tools for the Social Determinants of Health (11) as a template to organise the single determinants into broader, more easily comprehensible, and comparable, domains.

**FIGURE 2:**
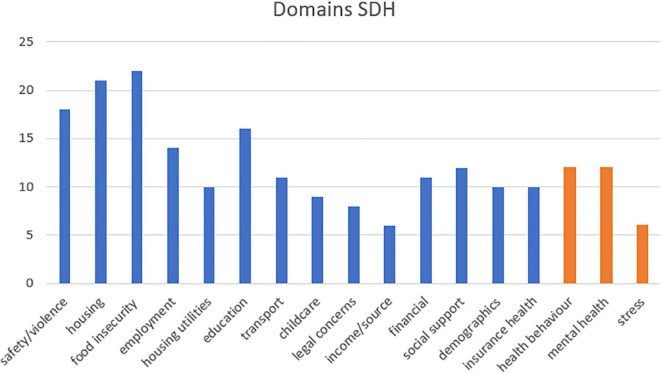
Domains of the Social Determinants of Health.

In the analysed literature we found all 15 domains previously revealed by Moen and colleagues (identified by blue bars in Figure 2). An exhaustive list of all determinants that we allocated to the single domains is in the Supplementary Material 4. The order of domains follows Moen and colleagues (11), arranged in descending frequency from the left. Moreover, our analysis of the included literature revealed three additional domains, “health behaviour,” “mental health” and “stress” (orange in Figure 2). We decided to add these three extra domains to classify determinants featuring in the analysed literature that could not be allocated to one of the domains that Moen and colleagues proposed.

The most commonly identified domains of Social Determinants of Health roughly replicated the findings of Moen and colleagues (11). That is, domains were in tendency more frequent the further they are on the left in the diagram. The three most prominent SDH domains were “food insecurity,” “housing,” and “safety/violence.” More than half of the papers referred to “education,” “employment,” “social support,” “health behaviour,” and “mental health.” Eleven of the 25 papers considered the domain “transport” and nine “child-care”.

### Social Determinants of Mental Health

**FIGURE 3:**
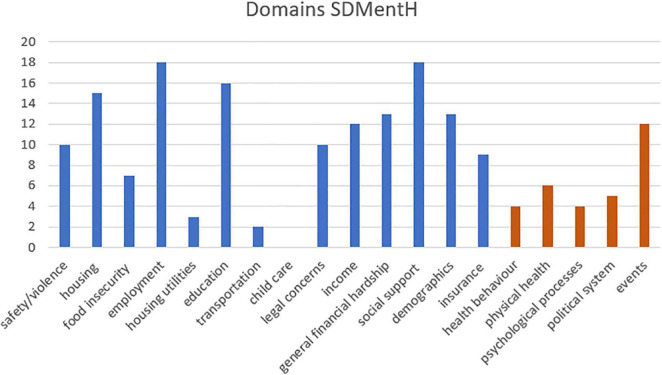
Domains of the Social Determinants of Mental Health.

We used the same template provided by Moen and colleagues (11) to structure the factors that were considered to be Social Determinants of Mental Health in the analysed literature, see Figure 3. Our analysis revealed five additional domains, depicted in orange in Figure 3, “health behaviour,” “physical health,” “psychological processes,” “political system,” and “events” (defined as time-specific events, like the loss of a loved-one, divorce, or an unintended pregnancy, Adverse Childhood Experiences, as well as more vaguely worded environmental, industrial events or emergency situations).

The literature on Social Determinants of Mental Health did not exactly fit the pattern of Moen and colleagues, in that the prominence of domains differed somewhat.

The most prominent domains for SDMentH highlighted in the current review were “employment,” “social support,” and “education.” More than half of the papers referred to “general financial hardship,” “housing” and “demographics.” Twelve papers considered “income” and “events”. Only two considered “transport” and none “child-care”.

***Questions 2 and 3: What epistemological assumptions underpin both constructs (SDH and SDMentH)? What does the literature tell us about potential pathways from Social Determinants of Health and Social Determinants of Mental Health to specific experiences of individuals?***

We conducted template analyses to answer these questions for underlying epistemologies and aetiological assumptions. Our analysis revealed seven themes that were deemed “useful, plausible and relevant to the purpose of the synthesis” (38): multifactorial multilevel models; interconnected and interdependent determinants; direct pathway mechanisms; stress as mediator; health behaviour as mediator; vicious cycles; role of perception and appraisal. In the process of developing the template, one of the initial themes (mastery, freedom, control) was removed because it did not bear relevance for the broader literature, and one theme (multifactorial multilevel model) was introduced, because it added to the conceptual model. These themes were not independent, and not every paper contained information on each theme. Themes varied in depth, from rather broad to specific themes, in line with guidelines for template analysis (39).

### Social Determinants of Health

#### Multifactorial Multilevel Model

All analysed papers conceptualised health by recognising multiple factors involved in aetiology. This conceptualisation was sometimes explicitly stated and, at other times implied. Moreover, health was explained as being socially determined on multiple levels. Thus, health is conceptualised by multiple factors on multiple levels. Single publications differed in the number of levels they distinguished and how these levels were named. For example, Suzy Barcelos Winchester (43) differentiated between individual-, household-, community-, cultural-, and context-level, whereas the Institute of Medicine (42) made distinctions more broadly in up- and downstream levels, as did Bourgois and colleagues (49). The remaining papers revealed multilevel conceptualisations more implicitly.

#### Interdependence and Interaction of Determinants

The majority of the analysed papers referred to the interdependence of different Social Determinants of Health. Some explicitly mentioned the interdependent dynamics of SDH (43, 47, 58), while most listed several exemplary associations. Most commonly, lists of associations between different determinants were explained with poverty, income or Socio-Economic Status (SES) as a starting point (43, 44, 61, 62). Other papers depicted interdependent determinants starting with gender (42, 63), or race (42, 43, 61), transport (64, 90), or employment (42, 90). Some of the associations will be outlined in more detail in the following themes.

#### Direct Pathway Mechanism

Several determinants were described as directly shaping (physical) health. Most often, toxins in the housing environment were understood to cause respiratory diseases or allergies directly (42, 43, 61, 64, 90). Furthermore, violence, utility needs and insufficient housing conditions were depicted as impairing physical health directly by causing injuries (42, 61, 90). Low income was indicated to cause poorer health (62) and adverse childhood experiences (ACEs) and intimate partner violence (IPV) to physically change brains (61) and cause mental disorders (42).

#### Stress as a Mediator

“Stress” was considered a determinant itself in several papers, but it was also conceptualised as a pathway mechanism through which social conditions affect health. Social conditions that lead to chronic stress, were considered especially detrimental for health (43, 46, 62). This stress was explained to affect individuals directly via biological pathways (HPA axis, neuroendocrine system, epigenetic changes) (43, 47, 62). Stress was also claimed to have indirect effects on health by motivating behaviour (42, 48).

#### Health Behaviour as Mediator

Health behaviour was, like stress, frequently considered to be a determinant itself and was additionally seen as one mechanism through which social determinants affect health (46, 47). Generally, behaviour that is considered to mediate between social conditions and health in the analysed literature can be divided into three types: violent behaviour (42, 63), lifestyle related behaviour (42, 43, 48, 58, 62) and behaviour directly related to health care management (42, 58, 61). An example for the violent behaviour is gender-specific risk taking (63). Lifestyle related behaviour is explained to mediate between social factors and physical health factors for example in the way of social isolation leading to poor health outcomes mediated through emotional eating (58). Behaviour directly related to health care management reifies for example in medication errors (61) or lacking use of preventative care measures (42).

#### Vicious Cycles

The dynamic between poverty and health was occasionally described as bidirectional, generating vicious cycles (42, 61, 62). Thus, low income was explained to directly deteriorate health outcomes, but on the other hand good health was considered a precondition of income (62). Hence, financial resources and health manifest cycles where an increase in financial resources leads to an increase in health and a decrease of available financial resources to a decrease in health.

#### The Role of Perception and Appraisal Processes

Perception and appraisal processes were only sporadically thematised in the literature on the Social Determinants of Health. One example of the consideration of perceptual processes in an illness development was the perceived support of God, which was conceptualised as a part of religious social capital that shapes coping behaviours (48). Moreover, shared beliefs were considered to enhance social religious capital (48). Poverty was considered to affect health, especially happiness, through valuation and world conceptualisation (62). It was also assumed that stress would be a genuine matter of perception and that perception would primarily be associated with mental health and self-reported health (42). Finally, the appraisal of cultural and normative markers such as accent and etiquette, was deemed to influence one’s opportunities and treatment (49).

### Social Determinants of Mental Health

#### Multifactorial Multilevel Model

All analysed papers presumed a multifactorial aetiology of mental health that is influenced by biological, social, psychological, environmental, genetic, and behavioural factors. Additionally, all papers explained SDMentH in a multilevel frame, more or less explicitly. Explicitly were the levels divergently categorised. Some only outlined two levels: an “individual and a societal/political” (70, 80), or “distal and proximal” (68, 76). Other papers differentiated between three or more levels, like the “national, local, and individual” (69), “macro level, community, family, and individual level” (77), “the socioeconomic, political and cultural level, the daily living conditions and the individual health-related factors” (71), “global-, macro-, exo- and micro-level” (84), “individual-, family-, community-, structural-, population-level” (66). More implicitly were multiple levels entailed by referring to “root-causes” (different levels implied by the botanic-metaphor) (70, 80), or the “causes of the causes” (since “cause” as an underlying mechanism again implies different levels) (89).

#### Interdependence and Interaction of Determinants

The vast majority of the analysed papers described the interdependent dynamics of different determinants of SDMentH. This interdependence was sometimes explicitly expressed (68, 88), accompanied with the remark that determinants must not be artificially separated, and that it would be important to understand how single determinants are related to each other (81). Some papers stated that single social determinants would contribute to a non-linear, dynamic interplay in a complex system (73, 79). Other papers did not theoretically comment on the interplay of single determinants but illustrated these dynamics more concretely in specific associations. Such webs of determinants were centred on SES (69, 71, 82, 83, 87), gender (65, 68, 69, 76, 85), race (78, 82, 87), age (66, 69, 82), or employment (72, 76, 77, 81).

#### Direct Pathway Mechanism

Social Determinants of Mental Health (SDMentH) were defined, by some, as the determinants that directly affect mental health (66, 72). However, the analysed papers barely depicted direct pathways. One of the few examples for direct causation was discrimination, which was described as having indirect (via poverty, employment, housing and so forth) as well as direct (via traumatisation) effects on mental health (77). Smoking, alcohol consumption and poor sanitation was claimed to have a direct impact on mental health through hormones and biological phenomena of addiction (69). Homelessness might be causally and thereby directly related to mental illness (88). And the built and natural environment was deemed to have direct as well as indirect effects on mental health, although only the indirect effects were exemplified (66). Furthermore, one model stated independent effects of gender, age, education, and income on mental health (85).

#### Stress as Mediator

Stress was considered to function as a mediator between social conditions and mental health in the majority of analysed papers (65, 69, 72–77, 79, 81, 84, 89). The pathway mechanism was most frequently described on a biological, hormonal and neuroendocrinal level (68, 72–74, 79, 82, 87, 89). The physiological stress-response system was considered to alter when acute stress is turning into chronic stress (65, 73, 75, 87). This affects the sensitivity to stressors (75) and behaviour (see next section). Furthermore, stress was also depicted as an indicator of any negative life events (78) and certain mental disorders themselves (75).

#### Health Behaviour as Mediator

Health behaviour was less commonly identified as a social determinant of mental as opposed to physical health (see domains). It was, however, occasionally described as a mechanism through which social determinants affect mental health. Behaviour associated with deteriorating mental health was conceptualised as stemming from restricted choices (74, 78, 80, 84) and resulting from stress (66, 73, 75, 80, 89). A family history of depression was hypothesised to affect the mental health of other family members through learned behaviours (69). More generally, risky and addictive behaviour was described as important in the development of mental health difficulties (75, 80, 85, 89). Furthermore, parental behaviour was described as affecting the mental health of children and being affected by social conditions (77, 88). However, it is also stated that health behaviours play a more significant role in the explanation of SDH mechanisms than for SDMentH (68).

#### Vicious Cycles

The relationship between social determinants and mental health in general was conceptualised as bidirectional (67, 69, 87). Various social determinants like discrimination, employment and limited economic opportunities were described to lead to poor mental health, while mental health problems vice versa often result in experiences of discrimination, unemployment, and decimated economic resources (87). Social determinants were therefore seen as consequences and conditions of mental health. Poverty and poor mental health, in particular, were explained as constituting vicious cycles (68, 71, 76), through increased costs of treatment and housing and reduced ability to work (69). Furthermore, behaviour and mental health were reported to be co-dependent (71) as were depression and deprivation (69).

#### The Role of Perception and Appraisal Processes

Many of the analysed papers emphasised the importance of perceptual processes. Perception and appraisal become particularly important in the translation of objective stressors to long term mental health impact (65, 68, 73, 75, 77, 84, 88). How objective stressors are appraised depends partly on the degree of perceived control an individual has (72, 77, 79, 82). In addition, perception is considered to be shaped by personality and behavioural factors (88), accessible resources (68), and social beliefs and norms (65, 70, 74, 75). Furthermore, the definition of mental illness and health itself is understood to be culturally formed and thereby a matter of appraisal (69, 76, 77). It was stated that resulting perceptions shape emotions (84) and behaviour (73, 75, 83). Moreover, it was argued that the appraisal of conditions poses a greater impact to an individual’s mental health than objective realities (73, 80, 83, 84, 87). Additionally, relative deprivation was considered more detrimental than absolute deprivation (80, 87). Appraisal on a more abstract level in the form of social norms was conceptualised to cause in a bidirectional interplay with public policies the distribution of opportunities and by that mental health (74).

***Question 4: How do SDH and SDMentH compare to each other?***

### Explicit Comparison in the Literature

Potential differences between SDH and SDMentH were, sporadically, directly addressed in the analysed papers. Some suggested that the two concepts are not necessarily distinct from each other (70), or even largely the same (74), but that SDMentH have been neglected to a great extent, and thereby less well understood (70). Others suggested that SDMentH and SDH differ on a number of features, although these features were not explicated (68). Furthermore, the construct of SDMentH was sometimes presented as an autonomous construct, without consideration of the relationship to SDH (72, 76). Papers that did address the relationship between SDH and SDMentH used the terms Social Determinants of Health and Social Determinants of Mental Health interchangeably (73, 79, 81). Posited arguments for this practice were the inextricable relationship between mental and physical health (66, 80, 82), and the claim that mental health is an integral part of the broader health (67, 86). Potential differences between SDH and SDMentH were often not considered (65, 83) and it was stated that a differentiation might obscure more than it would elucidate (87).

### Comparison Regarding the Considered Determinants

Overall, the range of determinants considered for the constructs SDH and SDMentH were similar. So were three domains (employment, education, housing) featuring in the top five of most frequently considered determinants for both constructs. Antipodal considerations of certain domains, domains considered highly relevant for one construct and irrelevant for the other, were rare. However, there were differences between SDH and SDMentH regarding their prioritisation of single domains. These differences pertained to the absolute number of papers considering a certain domain, as well as the relative position certain domains had in the hierarchy of the most prominent domains. “Social support” and “income” were much more frequently considered in the literature of SDMentH, both in the absolute number of papers considering the domains and in the relative position of the domains in relation to the other domains. On the other hand, “housing utilities,” “transport,” “health behaviour” and “childcare” were far more often represented in the SDH literature, again absolutely and relatively.

### Comparison on Basis of the Themes

#### Multifactorial Multilevel Model

Social Determinants of Health (SDH) and SDMentH were both conceptualised as multifactorial on multiple levels. Yet, little agreement and clarity existed regarding the number of levels and what these different levels were. Significant differences between SDH and SDMentH were not made apparent in this context.

#### Interdependence and Interaction of Determinants

The single determinants in the SDH as well as the SDMentH construct were depicted to be interconnected and interdependent in various forms. Starting points for retracing the interdependencies of determinants were for both constructs’ socioeconomic status, gender, race, or employment. There were no significant differences in this context.

#### Direct Effects of Determinants on Health

In the analysed literature, SDH was more often described to follow direct pathway mechanisms compared to SDMentH. One of the main identified sources of direct impact on health were toxins in the housing environment. This was only once suggested for the SDMentH and caveated by a call for supportive evidence in that instance (69). Furthermore, injuries as direct manifestations of social conditions and direct causes of poor health were not reported to have relevance in the SDMentH-context, unless trauma was seen as equivalent to injuries (77). Other direct pathways in the SDMentH context were asserted but not evidenced or illustrated; only indirect pathways were further exemplified (66). Thus, SDH and SDMentH differed regarding their consideration of direct pathway mechanisms, with SDH being more frequently explained to follow direct pathway mechanisms.

#### Stress as Mediator

Stress was described as a mediator for both, SDH and SDMentH. The turning point of acute to chronic stress was, in both conceptualisations, described as a pivotal moment in health deterioration. Stress was predominantly explained in biological terms for SDMentH and SDH, and deemed to partly affect health and mental health through the motivation of behaviour. In contrast to SDH, stress was not considered to be a determinant itself in the context of SDMentH. Other than that, there were no significant differences between SDH and SDMentH regarding the consideration of stress.

#### Health Behaviour as Mediator

Generally, health behaviour was construed to play a role for SDMentH and SDH. However, health behaviour as one determinant or explanation was more pervasive in SDH literature. Furthermore, the recognition of behavioural factors in SDMentH tended to be more specific, such as learned behaviour was determined to be relevant in the aetiology of depression or parenting styles for the mental health of children. Behaviour directly related to mental healthcare management did not crop up as a component in SDMentH. In sum, there were minimal differences between the role of behaviour in SDH and SDMentH.

#### Vicious Cycles

Both, SDH and SDMentH were claimed to reify partially in vicious cycles, especially between poverty and health/mental health. The literature on SDMentH outlined vicious cycles more often and more extensively. Thus, SDH and SDMentH did not differ in principle with respect to vicious cycles, but this pathway mechanism was of more prominence for SDMentH.

#### The Role of Perception and Appraisal Processes

Appraisal and perceptual processes played a minor role in SDH, contrary to SDMentH. Where perception was taken into account in the analysed SDH literature, the outcome was often in the mental health realm (42, 62) (also, more indirectly in Cohen-Silver et al. (48), because health remains vague in this publication, but the first author has a background in psychiatry and most cited studies dealt with depression or drug use). On the contrary, appraisal was a dominant component in SDMentH-conceptualisations. Perception and interpretation were described as primary driving forces of SDMentH, even more relevant than objective realities. Furthermore, the SDMentH literature dealt with the social and individual origins of perceptual and appraisal processes. Importantly the definition of what constitutes mental illness is more a matter of discussion, and by that of perspective and perception, contrary to the definition of physical health. Thus, SDMentH and SDH differed significantly in their heed to perception and appraisal.

## Discussion

### Main Findings

There is conceptual ambiguity regarding the relationship between the Social Determinants of Health and Social Determinants of Mental Health. Some consider these two public health constructs to be categorically different from each other (68). Others conceptualise SDMentH as a sub-construct of SDH (73). To date, no systematic research has investigated the literature on the relationship between SDH and SDMentH. The current scoping review addressed this gap by extracting the considered determinants in the literature of both constructs and using a template analysis to compare the theoretical conceptualisations of both constructs with each other.

### The Considered Determinants

Analysis of the 25 papers retrieved for each construct indicated some differences and many similarities regarding the determinants. Overall, the same determinants were described in both literature sets but differed in how they were prioritised. For example, the three determinants (-domains) most commonly identified for SDH were in tendency concrete in their nature (food insecurity; housing; safety/violence). At least food insecurity and housing can be seen as tangible categorical factors, a person has enough food or does not, has access to housing, or does not. The equivalents for SDMentH were on the other hand rather immaterial (employment; social support; education). All these factors are social constructions contrary to the physical realities of housing, food, and violence. Furthermore, of these factors social support and education are dimensional factors. Education like social support exists to degrees and on a spectrum. This contrast is important because it speaks to the differences between direct pathways and effects shaped by appraisal processes, discussed in more detail below.

In short, Social Determinants of Mental Health are often conceptualised to affect mental health filtered through the perception of individuals. It is in line with this finding that two of the three most frequently considered determinants for SDMentH are of a dimensional nature where it matters to what degree one feels socially supported or educated. The Social Determinants of Health are not theorised to affect health mediated through appraisal or perception, but to work through direct pathway mechanisms. This corresponds to all three most frequently considered determinants referring to physical realities and two of the three most frequently considered determinants of SDH being either absent or present, with not much room for debate. However, the juxtaposition of determinants most frequently considered should not be overinterpreted, as many social determinants (like housing) are particularly prevalent for both constructs. Moreover, how often a determinant was considered in the analysed literature is certainly one indicator of significance for the construct, but it is not the only one. It is striking that the mental health domain featured in nearly half (12 out of 25) of the analysed SDH-papers. This speaks to the high relevance of mental health as a component of, or as a condition for health, but it does not necessarily imply that SDMentH would be conceptualised as sub-construct of SDH. This is because it would be conceivable that interconnected constructs could function through different mechanisms for health or mental health contexts. The same observation holds for physical health as a Social Determinant of Mental Health (six out of 25 papers in the SDMentH literature). It seems reasonable to suppose that mental health is affected by our physical health, and vice versa, each could be a determinant of the other. It would be inappropriate therefore, to conclude that this dynamic relationship confirms that either one construct is a sub-component of the other.

### Similarities and Slight Differences Between the Constructs on Aetiological Grounds

The template analysis revealed seven interrelated patterns of theoretical explanations, or themes (36): multifactorial multilevel models; interconnected and interdependent determinants; direct pathway mechanisms; stress as mediator; health behaviour as mediator; vicious cycles; role of perception and appraisal.

Some of these themes exposed similarities between both constructs. Specifically, SDH and SDMentH were conceptualised in multifactorial and multilevel models, with interconnected determinants. Both constructs, are subject to some of the same potential logical flaws. In discussion of SDH and SDMentH, the interdependence of different determinants was regularly used as an explanation of a pathway mechanism. In fact, explaining the effect of certain determinants by pointing out that they are connected with other determinants is not an explanation, but merely an observation that could result in an “infinite causal regress” (91).

The remaining five themes revealed slight to more significant differences between SDH and SDMentH.

Health behaviour appeared both as a mechanism explanation and as a determinant for SDH and SDMentH. The prominent consideration of health behaviour as determinant in the analysed SDH-literature contradicts the call to focus on distal and fundamental determinants (92–95). Focusing on proximate factors such as health behaviours, as decontextualised from the distal determinants they have stemmed from, runs the risk of blaming the victims, rather than remedying existing health inequalities (96). Contrary to this, in the SDMentH-literature, health behaviour was more consistently contextualised in social conditions, which is in line with propositions on how to avoid a “lifestyle drift” (97, 98).

Stress was described as a pivotal mechanism in the translation of social condition into health for SDH and SDMentH. In particular the transition of acute to chronic stress was emphasised to be a critical aetiological moment. It is striking that SDH and SDMentH did not differ significantly in the consideration of stress. Because although stress is equivocally defined (99), it is widely agreed that stress is a phenomenon of appraisal and perception (100, 101). The prominent role stress takes in the analysed SDH-literature therefore contrasts with findings regarding “perception and appraisal,” because perception- and appraisal-processes were not explicitly elucidated to play an important role in the SDH context. The emphasis on stress might introduce the appraisal aspect more subtly into the SDH context. Some SDH-papers acknowledged the perception component of stress (42), but in the majority of SDH-papers this element remained unmentioned.

### More Significant Differences Between Both Constructs on Aetiological and Epistemological Grounds

It seems appropriate to discuss three themes in tandem (direct pathway mechanisms, vicious cycles, and role of perception and appraisal), as they conjointly tell one epistemological and aetiological story. SDH was frequently described to follow direct pathways, whereas SDMentH tended to be more often depicted in vicious cycles. Furthermore, SDMentH conceptualisations outlined perceptual and appraisal processes as particularly important in aetiologies. The differences between SDH and SDMentH in respect to the two themes “direct pathway mechanisms” and “role of perception” were especially unequivocal and indicate diverging epistemologies, which will be outlined in the following paragraphs.

Direct pathway mechanisms in the SDH context were for example explained in the following way: toxins in the housing environment causing respiratory diseases, or hazardous neighbourhoods leading to injuries, implying one given reality that has common repercussions. This aetiological stance, in which material objects such as housing, transport, food insecurity etc. affect health directly, can be subsumed under neo-materialism (98, 102). Neo-materialism is based on a positivist epistemology where “to measure is to know” (103). Also, the currently analysed SDH- literature supports this association of neo-materialism and positivism in at least three ways. Firstly, the assessment-literature can be seen as symptomatic of a field criticised for over-reliance on empirical research at the expense of theoretical work (104). Secondly, the relative preponderance of direct pathway explanations, that state that the material world directly affects health. Thirdly, the aforementioned three most frequently considered determinants of SDH, all of which are directly measurable and material (to a degree).

On the contrary to this direct, materialist perspective lies the focus on perception and appraisal in the conceptualisation of SDMentH. The dominant aetiological understanding for SDMentH is that social determinants affect mental health filtered through individuals’ perception, a stance in line with the assumptions of the psychosocial pathway (3, 105, 106). Within these suppositions of the psychosocial pathway, social capital and social support are considered to be the pivotal mediators and determinants of health (107, 108). This is consistent with social support being the most frequently considered domain for SDMentH in the present analysis, especially because determinants that have been subsumed in the social support domain could arguably also be classified as elements of social capital (see Supplementary Material 4). Importantly, the psychosocial pathway rests on a different epistemological foundation than neo-materialism. The focus on individuals’ perception questions that individual experiences necessarily match objective events, an epistemological stance accounted for under the umbrella term interpretivism (109, 110). One reification of this stance is critical realism (103) which has been explicated as the framework of SDMentH before (111, 112). According to this framework, assumptions, and experiences in the one given real world are always embedded in theoretical presumptions (113). In this context belongs the reappearing issue that the definition of mental health itself is changing and manmade.

Social Determinants of Mental Healths (SDMentH’s) focus on psychosocial pathways is not surprising. Mental health is defined by the WHO primarily in the context of realising potentials and coping with stressors; a stance that clearly implicates perception and appraisal. The ICD 11 defines mental, behavioural, or neurodevelopmental disorders as significant disturbances in cognition, emotional regulation, or behaviour—that is, with an emphasis on appraisal and perceptual processes. Hence, the dominance of the psychosocial pathway was to be expected. Furthermore, the pathway explanation of Social Determinants of Mental Health in vicious cycles is in line with postulations of critical realism that causality occurs within complex systems rather than linear constellations (110). Moreover, empirical evidence supports the hypothesis that neo-materialist explanations are not significant in the aetiology of mental illness (114).

Thus, SDH and SDMentH appear to differ in their fundamental epistemology and explanatory approach. A remark that has been implied 30 years ago already:

“Socio-economic inequalities in health cannot be wholly accounted for in terms of increased exposure to physical, chemical and organic dangers and dis- advantages. This is most obvious in those causes of death (such as suicide) and those illnesses (psychiatric and psychosomatic disorders) where conscious states of the person have an acknowledged causal or mediating role” (115).

However, it is without doubt that SDH is not only conceptualised in a neo-materialism-framework (116). Conversely, the epistemological conceptualisations of SDH are diverse (117) and reify especially in the vigorous debate around the income-inequality-hypothesis, which states that health deteriorates in dependence from the existing income inequality (118, 119). Most likely is that SDH affect health directly in part, and partly via psychosocial processes, that is, appraisal pathways and beliefs (120). The analysed literature provides support for this hypothesis by occasionally also considering the relevance of perception. And on the other hand, the analysed SDMentH literature does take some direct pathways into account. That means SDH and SDMentH are not distinctively delineated regarding the direct vs. perception mediated pathways/positivism vs. critical realism/materialist vs. psychosocial pathway. However, taking the findings of this review together, SDH is more strongly conceptualised in direct pathways and SDMentH is more strongly conceptualised in psychosocial pathways.

### Philosophical Consequences of Differentiating the Two Constructs From Each Other

Differentiating mental from physical illness, even only tentatively, has far reaching philosophical consequences because it implies a mind-body dualism (121, 122). The current review marks the role played by perception as the crucial dividing point between mental and physical health. This is consistent with the recognition that the mind-body problem becomes particularly difficult to solve in the face of subjective experiences (123) or qualia (124, 125), which has been called the hard problem of consciousness (126). Consciousness appears to be not fully reducible to states of the brain and thereby the body. In consequence, physicalism, the position that everything which exists is no more extensive than its physical properties and that the mind would therefore be fully retractable to the body, would need to be refused (123). Notwithstanding the hard problem of consciousness, it is almost routine to read that any kind, of dualism, assuming a categorical difference between mind and body, would be incompatible with a modern understanding of psychiatry (122, 127). Against this commonly shared opinion, naturalistic dualism recently emerged as an option compatible with the biopsychosocial model of modern psychiatry (128) and able to solve the hard problem of consciousness (20, 128). The naturalistic dualism after Chalmers posits only one (physical) substance but two kinds of properties, the physical properties and phenomenal properties. Phenomenal properties are understood as the subjective quality of experiences and consciousness, as fundamentally different from physical properties (126). Both properties are conceptualised to be interrelated through hitherto unrevealed psychophysical laws (128). Phenomenal properties are involved in physical and psychiatric conditions, but they are arguably more essential for mental health. This is in accordance with the findings of this review, where perception and appraisal processes (phenomenal properties) are of more importance in the aetiology of mental health compared to the aetiology of physical health. Delineating psychiatric from medical conditions on the basis of naturalistic dualism is neither philosophically uninformed anymore (20) nor indicating thought-absence in physicians [against the claims of Kendell that “two assumptions that have long since been abandoned by all thinking physicians, namely that mental disorders are disorders of the mind rather than the body, and that they are fundamentally different from other illnesses” (122)]. We explicitly place our findings in the philosophical framework of naturalistic dualism.

### Limitations

This review has a number of potential limitations. First, we compared literature on the assessment of SDH with literature on the theoretical groundwork of SDMentH. We chose this strategy for several reasons. The literature-scops of the two constructs are incommensurable, with the SDH-literature being too extensive to be fully comprehensible. To render this vast body of literature manageable we were forced to restrict the SDH literature, and focused specifically on material related to assessment, as this can be regarded as the nexus of theoretical frameworks and practical application. The data that is collected under a certain construct, in this case SDH, affects the understanding of the construct (23). Hence it is pivotal which theory is guiding the data-collection. Furthermore, measuring a construct serves as a performative definition and is, therefore, an application of underlying theory (27). A systematic literature search revealed that no comparable body of literature on the assessment of SDMentH exists. Therefore, we analysed theoretical papers, because a well-developed theory provides the condition of construct-validity and thereby a valid assessment method. Thus, to a degree the literature sets manifest different maturation-stages in a construct-development. It could be argued that the only valid inference of our comparison would regard the question whether there is an assessment method in accordance with the SDMentH-construct. This is a relevant question in its own right and it is certainly answerable, namely refusable, on the basis of the current review. However, we believe the current review yields further valid inferences, despite the limitations of our asymmetrical comparison. We did not compare genre-specific issues like structure of the papers or methodologies. Instead, we conducted a template analysis, that was flexible enough to involve both constructs and publication types. Theory papers and assessment papers represent manifestations of conceptualisations and can therefore both be consolidated to retrieve underlying aetiological and epistemological assumptions.

Second, the theories and assessment methods have not been analysed on the basis of quality, or compared, and have consequently all been considered coequally. Arguably, several indices would have been conceivable. Theories and assessment methods could have been appraised regarding their impact or measured, for example, in citations. Further criteria could have been the quality of the theories, as done by Bonell and colleagues (129), or the validity of assessment methods, or the publication type. None of these criteria have been applied because any choice would have been arbitrary to a degree, in excluding the remaining criteria. A governmental publication like that of the Victorian Health Promotion Foundation (71) might not have been cited very often, but it directly shaped the policies of Australia, with 25 million inhabitants. Impact is therefore in this context not unambiguously gaugeable. Moreover, the diverging foci of the two literature sets hampered a shared index (theory quality vs. psychometric properties). Importantly, however, a critical appraisal of the reviewed literature is not an obligatory part of a scoping review (24).

Third, the confinement of SDH-literature to assessment literature brought the advantage that considered determinants were readily enlisted, but it also meant that aetiologies and epistemologies were not always the main foci of the papers.

Fourth, although there were no restrictions on publication date, language was restricted to German and English, which might have resulted in relevant papers being excluded and a potential selection bias. However, both constructs are as public health issues internationally discussed in English. Therefore, we deemed the selection bias to be not too significant.

Fifth, the literature search scheme retrieved initially a much higher number of results for SDMentH compared to SDH. This might indicate a higher sensitivity of the SDMentH-search strategy (and by that matter definitely a higher specificity of the SDH-search strategy considering the number of eligible papers). The potentially decreased sensitivity has been accepted because several recent SDH-assessment tool reviews offered readymade comparative selections of papers.

Sixth, the seven revealed themes are not claimed to cover the constructs of SDH and SDMentH exhaustively. Both constructs are highly complex and too multifaceted to be all-encompassing subsumed in a few themes. Furthermore, the applied method of Pound and Campbell (38) does not demand an exhaustive depiction, but only to synthesise what was deemed “useful, plausible and relevant to the purpose of the synthesis.”

### Implications

The findings of this review have several implications for future research and give first indications for policy making and mental health care. First, it is important and reassuring to confirm that the considered determinants of SDH are by and large the same as SDMentH. That means that taking actions to improve these determinants promises to improve physical as well as mental health. This double benefit of single measures can provide a convincing argument in a policy remit that is guided by cost-benefit analyses.

However, the current review also revealed differences between the pathway mechanisms of the two constructs, differences that have several potential consequences. The perception or appraisal of certain social factors appears to be more important in the causal pathways leading to mental health problems compared to physical health, while physical health appears to be mainly driven by the factual presence or absence of these certain social factors. This again supports the development of a specific assessment method for the SDMentH, potentially based on the current review.

There is a danger that an emphasis on individual appraisal processes could limit political and structural opportunities and responsibilities to tackle health inequalities (98) because it individualises structural problems. However, we argue that the perception of social factors and stressors is not simply a willed, idiosyncratic, act, but is rather itself also socially shaped. Conceptualising the psychosocial pathway as driven by perceptions is not hindering political remedies but even has the potential to enable additional structural avenues. The juxtaposition of two studies should illustrate our point. In 2003, Artazcoz and colleagues found in a dataset from 1994 that being unemployed had a greater impact on mental health for men compared to women in Catalonia (130). Importantly, the female work participation rate in Catalonia was low compared to other western states at this time and classical gender roles were very prevalent, with men being meant to provide for families. Aydiner-Avsar and colleagues examined the same association again in 2019 in the United States and found no difference between the genders with respect to the impact of being unemployed on mental health (131). They explain their finding with the dual breadwinner model, the societal norm that men and women are equally responsible to earn money. This norm stands in contrast to the traditional gender roles that prevailed in the dataset from 1994 in Catalonia (130). The meaning of a stressor like unemployment, the way one perceives it, is consequently not a purely individual question but also shaped by macro factors like societal norms (101).

We would like to call these factors the Social Determinants of Perception. Political or educational interventions might positively influence such Social Determinants of Perception and therefore support public health measures rather than impede them. Examples could include trying to support a changed beauty ideal that would be less prone to resulting in overly critical and unhealthy self-perception or trying to establish a more positive failure-culture that affects how individuals appraise their own successes and failures. Moreover, measures could be taken to tackle the mental health stigma, which eventually might trickle down to individuals and how they perceive their own mental health problems, potentially changing their help seeking behaviour. We do not claim that these interventions would be new. But they can show that an assumed psychosocial pathway for Social Determinants of Mental Health is not necessarily limiting structural and political interventions but, on the contrary, has the potential to enabling new ones. In line with the assumptions of critical realism, we would argue that interventions are needed to tackle both the Social Determinants of Mental Health and the Social Determinants of Perception, that is the objective realities and the way we make sense of them. Unfortunately, the “structural and cultural origins of meaning”, what we described as the Social Determinants of Perception are still understudied (101). More research is needed into structural differences in the perception of the same events and conditions. Coming with this, more research is needed into potential interventions aiming at the Social Determinants of Perception.

## Conclusion

This scoping review compared the construct of SDH with the construct of SDMentH. Both constructs considered by and large the same determinants, although in different prioritisation. A template analysis revealed seven common themes regarding aetiological and epistemological assumptions. SDH and SDMentH resembled in that both were conceptualised in multilevel models, and that stress was declared to be a mediator between social conditions and health outcomes. Furthermore, both constructs were akin in describing health behaviour as mediator and to outline some dynamics in vicious cycles, even though there were minor differences regarding these themes. Significant differences manifested regarding the considerations of “direct pathways” and the “role of perception”. SDH was tendentially described to follow direct pathways on a (neo)-materialism-epistemological foundation. The SDMentH concept on the contrary rested preponderantly on more psychosocial pathways, considering social determinants affecting mental health mediated through perception. This implies significant differences on epistemological and aetiological grounds, in tendency. Hence, this scoping review indicates that SDMentH is distinct from SDH. This might yield relevant implications for policy and prevention planning and eventually also for treatment.

## Author Contributions

FH has carried out the data collection and analysis and wrote the manuscript. MS screened a selection of the manuscript. PK, MS, and ST edited the manuscript. All authors contributed to the article and approved the submitted version.

## Conflict of Interest

The authors declare that the research was conducted in the absence of any commercial or financial relationships that could be construed as a potential conflict of interest.

## Publisher’s Note

All claims expressed in this article are solely those of the authors and do not necessarily represent those of their affiliated organizations, or those of the publisher, the editors and the reviewers. Any product that may be evaluated in this article, or claim that may be made by its manufacturer, is not guaranteed or endorsed by the publisher.
