# Supplementary material for: A Scoping Review and Narrative Synthesis Comparing the Constructs of Social Determinants of Health and Social Determinants of Mental Health: Matryoshka or Two Independent Constructs?
Source: Front Psychiatry. 2022 Apr 14;13:848556. doi: 10.3389/fpsyt.2022.848556 (PMC9046700; doi:10.3389/fpsyt.2022.848556)
Supplement: Supplementary file 1 [file Data_Sheet_1.ZIP › Supplementray material 4.docx]

**Single determinants that were subsumed in the domains**

**Social Determinants of Mental Health**

**Safety/ violence**: discrimination/ physical, emotional, verbal abuse/ domestic violence/insecurity and powerlessness/ violence and instability/ exposure to violence, conﬂict, and war in childhood or adulthood

**Housing**: home environment/ housing/ lack of housing/ housing, and community infrastructure

**Employment**: work conditions/ unemployment/ occupation/ work & meaningful engagement

**Food insecurity**: food insecurity

**Education**: Education/ early years care and education provision, schools

**Housing utilities**: energy/ poor sanitation and built environment/ water

**Transportation**: transportation

**Legal concerns**: forced separation/ institutionalization/ migration status/ incarceration of household member/ increased migration/ dislocation/ The Asylum-Seeking Process/ mass incarceration and poor relations between law enforcement and communities/ human rights

**Income**: income/ income inequality/ national income

**General financial hardship**: financial resources/ debt/ SES/ poverty/ subjective financial strain

**Social support**: social inclusion/ social connection/ marital status/ social capital/ poor community cohesion/ social interactions among friends, peers, family members and neighbourhood/ social isolation/ divorce and romantic break up/ family relationships/ social network/ marital discord/ inadequate organisational support/ elder abuse

**Demographics**: gender/ age/ race/ethnicity/ sexuality

**Insurance health**: access to resources/ care/ health system and health policy

**Health behaviour**: unhealthy lifestyle/ Smoking/ alcohol drinking/ diet/ physical activity

**Physical health**: genetic risk/ parent health/ physical health status/ disability

**Psychological process**: stress/ psychological factors/ cognitive abilities/ poor resilience/ self-efficiency/ attributional style/ identity confusion/ work stress and burn out/ maternal mental disorder/ neighbourhood safety fears

**Political system**: democracy/ lack of participation/ welfare state/ political freedom/ unstable policy environment/ governance

**Events**: loss of someone, a breakup or divorce, or an unintended pregnancy, Adverse Childhood Experiences, or more vaguely worded environmental, industrial events or emergency situations

**Social Determinants of Health**

**Safety/violence**: discrimination/ exposure to violence/ unsafe neighbourhood/ neighbourhood adversity (Crime, violence, domestic abuse, and poverty/ discriminatory practices (institutional racism, discriminatory practices in medical treatment, and daily discrimination)/ interpersonal safety/ abuse/ safety/ firearm exposure

**Housing**: housing stability/ homelessness/ housing with whom/ renting/owning/ household amenities/ housing costs/ residential address

**Food insecurity**: nutritious eating habits/ worries of food insecurity/ Food insecurity (fast food restaurants, convenience stores, and food deserts/ food pantries (quality of them)/ CFO evaluation

**Employment**: occupation status/ unemployment/ educational and employment opportunities

**Housing utilities**: access to basic needs near your home/ lack of heat/ utility needs

**Education**: educational attainment/ learning style/ literacy/ parental health literacy

**Transportation**: transportation

**Childcare**: lack of after school activities/ childcare/ child support and custody

**Legal concerns**: incarceration status/ refugee status/ civic engagement/ legal problems/ Medical-Legal Partnership professional

**Low income**: low income

**General Financial Hardship**: SES/class/ poverty

**Social support**: social isolation/ poor social support/ family social support/ social network and trust/ Marital status

**Demographics**: sexual orientation/ gender/ culture/ born in- or outside of the states/ race/ ethnicity

**Insurance health**: healthcare access/ health insurance

**Health behaviour**: nutritious eating habits/ proper hydration/activities: how many days & how long at a time/ disengaged smoking or illicit drug use/ health status and health behaviours/ alcohol use/ tobacco use/ exposure/ Sense of healthy or unhealthy behaviours/ Safety precautions: seatbelts, helmets, firearms, street violence

**Mental health**: coping strategies/ life satisfaction/ psychosocial factors/ depression/ trust

**Stress**: stress
