# Supplementary material for: A Scoping Review and Narrative Synthesis Comparing the Constructs of Social Determinants of Health and Social Determinants of Mental Health: Matryoshka or Two Independent Constructs?
Source: Front Psychiatry. 2022 Apr 14;13:848556. doi: 10.3389/fpsyt.2022.848556 (PMC9046700; doi:10.3389/fpsyt.2022.848556)
Supplement: Supplementary file 1 [file Data_Sheet_1.ZIP › Supplementary material 1.docx]

| **Criteria** | **Inclusion** | **Exclusion** |
| --- | --- | --- |
| SDH | Reports on assessment of the general SDH, or the assessment of only one determinant if it is described as one representative of the broader SDH-concept. The term “Social Determinants of Health” is mentioned. It is irrelevant whether it is spelled in capitals or not. | One or more determinants are discussed but not clearly embedded in a wider SDH-concept |
| Health | Deals with general human health | Deals with the health of animals, or only with specific diseases |
| Assessment | Reports the generation of an assessment method, or with the theoretical underpinnings of the general task of developing SDH-assessment methods, of one or more determinants | Assessing the SDH is only cursorily covered or pure feasibility studies/ studies applying assessment tools that had been developed somewhere else/ quality improvement study/ comments on the practical establishment of SDH-assessment methods/ primarily about addressing the SDH |
| Assessment level | Deals with the assessment of SDH at the individual level | The assessment happens at any other level |
| Theoretical embedding | The concept of SDH is explained or it clearly refers to literature that does so | No theoretical contextualisation |
| Determinants/ population/ health | Claims general validity | At least two of the three elements are restricted |
| Publication date | Until February 2020 |  |
| Publication type | Peer-reviewed articles, editorials, governmental publications  Empirical and discursive, dissertations | Meeting abstract, poster, review of existing assessment-tools, pure assessment tools authors will be contacted for any publications providing a broader contextualisation |
| Language | English, German | Any other |
| Methods | No restrictions, thus qualitative, quantitative or mixed methods |  |
| **Criteria** | **Inclusion** | **exclusion** |
| SDMentH | The term “Social Determinants of Mental Health” is mentioned. It is irrelevant whether it is spelled in capitals or not. | The term is not mentioned |
| Framework | Clearly elaborates on an original framework, i.e. describe mechanisms, determinants, strategies. Could serve as the basis for an assessment-tool-development to ensure construct validity. | No clear description of framework/theory/model, only mentions; only describes elements/parts |
| Model | Model as in theoretical elaboration in line with Booth and Carroll’s guidance (27) | Model as economic model |
| Mental health | There is consideration of how factors determine mental health/ have an influence on mental health | Only considers physical health, or has wellbeing or life satisfaction as outcome |
| Determinants/ population/ mental health | Claims general validity | At least two of the three elements are restricted |
| Language | English or German | Every other language |
| Publication date | Until February 2020 |  |
| Study type | No restrictions |  |
| Publication type | Peer-reviewed articles, editorials, governmental publications  Empirical and discursive, dissertations | Meeting abstract, poster  authors will be contacted for any publications for a broader contextualisation |
| Methods | No restrictions, thus qualitative, quantitative or mixed methods |  |

*Table 1 Inclusion and exclusion criteria*
