# Supplementary material for: A Scoping Review and Narrative Synthesis Comparing the Constructs of Social Determinants of Health and Social Determinants of Mental Health: Matryoshka or Two Independent Constructs?
Source: Front Psychiatry. 2022 Apr 14;13:848556. doi: 10.3389/fpsyt.2022.848556 (PMC9046700; doi:10.3389/fpsyt.2022.848556)
Supplement: Supplementary file 1 [file Data_Sheet_1.ZIP › supplementary material 2.docx]

| **Title** | **Year** | **Authors** | **Country** | **Background** | **Publication** | **Focus** | **Multiple vs single determinants** | **Restriction to population** |
| --- | --- | --- | --- | --- | --- | --- | --- | --- |
| Social Determinants of Health Assessment Tool: Implications for Healthcare Practice | 2019 | Barcelos Winchester, Suzy | USA | Social  work | Peer review | Particular instrument development | Multiple | None |
| Implementing thrive, an EHR-based screening and referral system to address Social Determinants of Health (SDOH) in an urban safety net hospital | 2019 | De la Vega, Buitron et al. | USA | Medicine/ social work | Peer reviewed | Particular instrument development + feasibility study | Multiple | None |
| Developing a screening tool to recognise Social Determinants of Health in Australian clinical settings | 2018 | Brown-Yung, Kathryn  Freeman, Toby  Battersby, Malcom W.  McEvoy, Doug R.  Baum, Fran | Australia | Health, society and equity/ psychiatry | Peer reviewed | Particular instrument development | Multiple | None |
| Development and validation of the Social Determinants of Health Questionnaire and implications for “Promoting Food Security and Healthy Lifestyles” in a complex urban food ecosystem | 2018 | Gadhoke, Pretty  Pamberton, Salome  Foudeh, Ava  Brenton, Barrett P. | USA | Pharmacy and health science/ anthropology | Peer reviewed | Particular instrument development + validation | Food insecurity | None |
| Developing electronic health record (EHR) strategies related to health center patients' Social Determinants of Health | 2017 | Gold, Rachel  Cottrell, Erika  Brunce, Arwen  Middendorf, Mary  Hollombe, Celine  Cowburn, Stuart  Mahr, Peter  Melgar, Gerardo | USA | Family health | Peer reviewed | Particular instrument development | Multiple | None |
| Family fIRST, an Interactive Risk Screening Tool for Families in a SchoolBased Pediatric Clinic: A Look at Feasibility and Pilot Data | 2017 | Cohen-Silver, Justine  Laher, Nazeefah  Freeman, Sloane | Canada | Paediatric | Peer reviewed | Particular instrument development + feasibility study | Multiple | Children |
| Standardized Screening for Health-Related Social Needs in Clinical Settings The Accountable Health Communities Screening Tool | 2017 | Billioux, Alexander  Verlander, Katherine  Anthony, Susane  Alley, Dawn | USA | Medicare and Medicaid service | Peer reviewed | Particular instrument development | Multiple | None |
| Addressing Social Determinants of Health in a Clinic Setting: The WellRx Pilot in Albuquerque, New Mexico | 2016 | Page-Reeves, Janet  Kaufman, Will  Bleecker, Molly  Norris, Jeffrey  McCalmont, Kate  Ianakieva, Veneata  Ianakieva,Dessislava  Kaufman,  Arthur | USA | Family and community service | Peer reviewed | Particular instrument development + feasibility study | Multiple | None |
| PRAPARE implementation and action toolkit | 2019 | National Association of Community Health Centers, Inc., Association of Asian Pacific Community Health Organizations, and the Oregon Primary Care Association | USA | Not clear | By NGO | Particular instrument development + implementation and action tool kit | Multiple | None |
| Surveillance and screening for Social Determinants of Health: the medical home and beyond | 2016 | Garg, Arvin  Dworkin, Paul H. | USA | Paediatrics | Peer reviewed | View point | Multiple | None |
| Screening for Social Determinants of Health Among Children and Families Living in Poverty: A Guide for Clinicians | 2016 | Chung, Esther K. et al. | USA | Paediatrics | Peer reviewed | Tool development guidelines | Multiple | None |
| A Randomized Trial on Screening for Social Determinants of Health: the iScreen Study | 2014 | Gottlieb, Laura  Hessler, Daniele  Long, Dayna  Amaya, Anais  Adler, Nancy | USA | Family and community medicine/ paediatrics | Peer reviewed | Particular instrument development + feasibility study | Multiple | None |
| Religious social capital: Its measurement and utility in the study of the Social Determinants of Health | 2011 | Maselko, Joanna  Hughes, Cayce  Cheney, Rose | USA | Psychiatry and behavioural science | Peer reviewed | Particular instrument development + validation | Multiple | None |
| Issues in the measurement of Social Determinants of Health | 2008 | Mooney, Gavin  Fohtung, Nubong G. | South Africa | Health economics | Peer reviewed | Discussion paper | Poverty | None |
| Improving the Management of Family Psychosocial Problems at Low-Income Children’s Well-Child Care Visits: The WE CARE Project | 2007 | Garg, Arvin  Butz, Alene M.  Dworkin, Paul H.  Lewis, Rooti A.  Thompson, Richard E.  Serwint, Janet R. | USA | Paediatrics/ medicine, biostatistics | Peer reviewed | Particular instrument development + feasibility study | Multiple | None |
| Defining and measuring gender: a Social Determinant of Health whose time has come | 2005 | Phillips, Susan P. | Canada | Family medicine | Peer reviewed | Discussion paper | Gender | None |
| The EveryONE Project Advancing health equity in every community/ Social Determinants of Health guide to social needs screening tool and resources | 2018 | American Academy of Family Physicians | USA | Family physicians | NGO | Particular instrument development | Multiple | None |
| Capturing Social and Behavioral Domains and Measures in Electronic Health Records | 2014 | Institute of Medicine | USA | Social and behavioral scientists and clinical and public health practitioners and information technology (IT) experts | Governmental institution | Particular instrument development | Multiple | None |
| Revisiting the Social History for Child Health | 2007 | Kenyon, Chén  Sandel, Megan  Silverstein, Michael  Shakir, Alefiya  Zuckerman, Barry | USA | Paediatrics | Peer reviewed | guidance on obtaining an SDH-focused social history | Multiple | Children |
| Structural Vulnerability: Operationalizing the Concept to Address Health Disparities in Clinical Care | 2017 | Bourgios, Phillipe  Holmes, Seth M.  Sue, Kim  Quesada, James | USA | Social medicine/ anthropology | Peer reviewed | Particular instrument development + feasibility study | Multiple | None |
| Screening for Toxic Stress Risk Factors at Well-Child Visits: The Addressing Social Key Questions for Health Study | 2018 | Selvaraj, Kevitha et al. | USA | Paediatrics | Peer reviewed | Particular instrument development + feasibility study | Multiple | None |
| Rethinking the Social History | 2014 | Behforouz, Heidi L.  Drain, Paul K.  Rhatigan, Joseph J. | USA | Global health equity | Peer reviewed | Commentary/ viewpoint | Multiple | None |
| Development of a Tool to Identify Poverty in a Family Practice  Setting: A Pilot Study | 2011 | Brcic, Vanessa  Eberdt, Caroline  Kaczorowski, Janusz | Canada | Family practice | Peer review | Particular instrument development + feasibility study | Multiple | None |
| Using Standardized Social Determinants of Health Screening Questions to Identify and Assist Patients with Unmet Health-related Resource Needs in Health and Human Services | 2018 | Sokol, Rebeccah et al. | USA |  | North Carolina Department of Health and human service | Particular instrument development | Multiple | None |
