# Supplementary material for: A Scoping Review and Narrative Synthesis Comparing the Constructs of Social Determinants of Health and Social Determinants of Mental Health: Matryoshka or Two Independent Constructs?
Source: Front Psychiatry. 2022 Apr 14;13:848556. doi: 10.3389/fpsyt.2022.848556 (PMC9046700; doi:10.3389/fpsyt.2022.848556)
Supplement: Supplementary file 1 [file Data_Sheet_1.ZIP › supplementary material 3.docx]

| **Title** | **Year** | **Authors** | **Country** | **Background** | **Publication** | **Focus** | **Multiple vs single determinants** | **Restriction to population** |
| --- | --- | --- | --- | --- | --- | --- | --- | --- |
| Victorian Health Promotion Foundation/ A Plan for Action 2005-2007 Promoting Mental Health and Wellbeing | 2005 | Mental Health and Wellbeing Unit Victorian Health Promotion Foundation | Australia | Not clear | Governmental report | Framework for the promotion of mental health and wellbeing | multiple | None |
| Social Determinants of Mental Health: the missing dimensions of mental health nursing? | 2007 | Lauder, W.  Kroll, T.  Jones, M. | UK | Nursing | Peer reviewed | Exemplary exploration of SDMentH from a mental-health-nursing-perspective | Multiple | None |
| Democracy: the forgotten determinant of mental health | 2007 | Wise, Marylin  Sainsbury, Peter | Australia | health promotion, public health | Peer reviewed | Exploration of one specific SDMentH aiming to stimulate debate | Single (democracy) | None |
| The role of public health in addressing racial and ethnic disparities in mental health and mental illness | 2010 | Primm, Annelle B. | USA | Psychology | Peer reviewed | Model proposition + policy recommendation | Racial and ethnic disparities | None |
| Social determinants and depression in later life | 2012 | Cross-Denny, Bronwyn | USA | Social work | PhD | Quantitative study | Multiple | Later life |
| Social Determinants of Mental Health | 2014 | Allen, Jessica  Balfour, Reuben  Bell, Ruth  Marmot, Michael | South Africa/ UK | Psychiatry/ global mental health | Book chapter | Unspecified review | Multiple | None |
| Social determinants of refugee mental health | 2014 | Mawani, Farah N. | Canada | Public health | Book chapter | Unspecified review | Multiple | Refugees |
| The Social Determinants of Mental Health: An overview and call to action | 2014 | Shim, Ruth  Koplan, Carol  Langheim, Frederick J.P.  Manseau, Marc W.  Powers, Rebecca A.  Compton, Michael T. | USA | Public health | Peer reviewed | Unspecified review + policy recommendations | Multiple | None |
| Does employment security modify the eﬀect of housing aﬀordability on mental health? | 2016 | Bentley, Rebecca  Baker, Emma  LaMontagne, Anthony | Australia/ UK | Health equity/ architecture | Peer reviewed | Model proposition + quantitative test | Housing and employment security | None |
| The Social Determinants of Mental Health | 2016 | Sederer, Lloyed I. | USA | Epidemiology | Peer reviewed | Comment | Multiple | None |
| Impact of globalisation on mental health in low- and middle-income countries | 2016 | Sharma, Sagar | India | Psychology | Peer reviewed | Review | Multiple, starting from globalisation | Low and middle-income |
| Assessing Social Determinants of Severe Mental Illness in High-risk Groups | 2014 | Sun, Qi | USA | Sociology | PhD | Model proposition + quantitative test | Multiple | Severe mental illness |
| Social Determinants of Mental Health | 2017 | Carod- Artal, Francisco Javier | UK/ Spain | Neurology | Book chapter | Unspecified review | Multiple | None |
| Developing a Health Inequalities Approach for Mental Health Social Work | 2017 | Karban, Kate | UK | Social work | Peer reviewed | Unspecified review | Multiple | In the context of social work |
| Men's Mental Health: Social Determinants and Implications for Services | 2018 | Affleck, William  Carmichael, Victoria  Whitley, Rob | Canada | Psychiatry/ mental health | Peer reviewed | Unspecified review | Multiple | Men |
| Social Determinants of Mental Health: Where We Are and Where We Need to Go | 2018 | Alegría, Margarita  NeMoyer, Amanda  Falgàs Bagué, Irene  Wang, Ye  Alvarez, Kiara | USA | Disparity research/ psychiatry/ health care policy | Peer reviewed | Unspecified review | Multiple | None |
| The Social Determinants of Refugee Mental Health in the Post-Migration Context: A Critical Review | 2018 | Hynie, Michaela | Canada | Psychology | Peer reviewed | Unspecified review | Multiple | Refugees |
| Social determinants of mental disorders and the Sustainable Development Goals: a systematic review of reviews | 2018 | Lund, Crick et al. | South Africa/ UK | Public mental health | Peer reviewed | Model proposition + review + policy recommendations | Multiple | None |
| A theory of public wellbeing | 2019 | Fisher, Matthew | Australia | Health, society and equity | Peer reviewed | Model proposition | Multiple | None |
| “MACRO MATTERS”: THE RELATIONSHIP OF SOCIOCULTURAL FACTORS AND MENTAL HEALTH across countries | 2017 | Scholten, Saskia | Germany | Psychology | PhD | Model proposition + quantitative test | Multiple (Wealth/ freedom/ justice) | None |
| Mental disorders: equity and social determinants | 2010 | Patel, Vikram et al. | India/ South Africa/ UK | Psychiatry/ global mental health | Book chapter | Unspecified review | Multiple | None |
| The Social Determinants of Mental Health: implications for research and health promotion | 2010 | Fisher, Matthew  Baum, Fran | Australia | Health, society and equity | Peer reviewed | Unspecified review + policy recommendations | Multiple | None |
| Determinants of mental health and self-rated health: a model of socioeconomic status, neighborhood safety, and physical activity | 2014 | Meyer, Oanh L.  Castro-Schillo, Laura  Aguilar-Gaxiola, Sergio | USA | Psychiatry | Peer reviewed | Model proposition + quantitative test | Neighbourhood safety, SES, physical activity | None |
| The Social Determinants of Mental Health | 2015 | Compton, Michael T.  Shim, Ruth S. | USA | Psychiatry | Peer reviewed | Model proposition | Multiple | None |
| Social Determinants of Mental Health | 2014 | Lund, Crick  Stansfeld, Stephen  De Silva, Mary | UK | Health equity | Peer reviewed | Overview | Multiple | None |
